# A comparison of the clinical effectiveness of pretreatment olive oil administered as drops versus spray prior to earwax removal by microsuction in adults: a protocol for a cluster randomised control trial

**DOI:** 10.1186/s13063-025-09378-5

**Published:** 2025-12-18

**Authors:** Linor Llwyd Jones, Kevin Munro, Jane Wild

**Affiliations:** 1 Betsi Cadwaladr University Health Board, Bangor, UK; 2 The University of Manchester, Manchester, UK

**Keywords:** Earwax, Wax removal, Olive oil, Microsuction, Wax softener, Cerumenolytic

## Abstract

**Background:**

Excessive earwax causes unwanted symptoms such as hearing loss, tinnitus, discomfort and changes in the quality of one’s own voice. The NHS Audiology Wax Removal Service in North Wales recommends the use of olive oil as a pretreatment wax softener administered as either drops or spray prior to microsuction. For one in four patients microsuction is unsuccessful at the first attempt. Anecdotal evidence suggests that the administration of olive oil as a spray is a more effective pretreatment. This trial aims to explore whether administering olive oil pretreatment softener is more effective when administered as drops or spray.

**Methods:**

This two-arm cluster randomised control trial will be conducted within the existing NHS Audiology wax removal service in North Wales from January to July 2025. This pragmatic trial involves 26 NHS GP practices (clusters) and compares the administration methods of olive oil pretreatment via drops and spray. A sample size of 1,742 participants (67 in each of the 26 practices) was calculated as sufficient to determine a clinically significant difference. Presumed consent is used for all eligible patients seen within the wax removal service. Patients will be advised to source and self-administer three drops or sprays of olive oil for seven days before attending a wax removal microsuction appointment with an audiology practitioner where routine anonymized data will be collected. The primary outcome is whether wax removal is successful, as assessed via visual examination. The secondary outcomes include: improvements in self-reported symptoms, the amount of residual wax following microsuction and the number of adverse events. Statistical analyses will be conducted with an intention-to-treat design to compare outcomes between the groups.

**Discussion:**

This RCT aims to investigate whether administering olive oil as a pretreatment wax softener via drops or spray affects the outcome of wax removal. The outcome of this trial will inform future recommendations to patients and improve service effectiveness and efficiency by reducing the need for repeat visits. The results will be shared nationally and used to inform national guidance on wax removal.

**Trial registration:**

ISRCTN, ISRCTN28211073. Registered 23 December 2024, https://www.isrctn.com/ISRCTN28211073?q=earwax&filters=&sort=&offset=1&totalResults=3&page=1&pageSize=10.

**Supplementary Information:**

The online version contains supplementary material available at 10.1186/s13063-025-09378-5.

## Administrative information

**Table:** 

Title {1}	A comparison of the clinical effectiveness of pretreatment olive oil administered as drops versus spray prior to earwax removal by microsuction in adults: a protocol for a cluster randomised control trial
Trial registration {2a and 2b}	ISRCTN28211073
Protocol version {3}	20th November 2024 Version 2.2
Funding {4}	None – trial run within existing clinical service
Author details {5a}	Linor Llwyd Jones, Principal Clinical Scientist, Audiology, Betsi Cadwaladr University Health Board Linor.ll.jones@wales.nhs.uk 03000 843 864 Prof. Kevin J Munro, Ewing Professor of Audiology, The University of Manchester Kevin.j.munro@manchester.ac.uk 0161 275 8678 Jane Wild, Consultant Clinical Scientist, Audiology, BCUHB jane.wild@wales.nhs.uk 03000 843,863
Name and contact information for the trial sponsor {5b}	Laura Longshaw, Deputy Research & Development Manager, Betsi Cadwaladr University Health Board Laura.longshaw@wales.nhs.uk 03000 856 766
Role of sponsor {5c}	Sponsor has the overall responsibility for ensuring that proportionate, effective arrangements are in place to set up, run and report this research study. In addition to being responsible for monitoring and audit activities, the Sponsor will advise the Chief Investigator on the study design, collection, management, analysis and report writing in accordance with our Standard Operating Procedures for Research

## Introduction

### Background and rationale {6a}

Earwax is a naturally occurring substance that is produced within the ear and forms part of the ear’s self-cleaning and protective mechanism. It achieves this by trapping dust and dirt and removing them from the ear canal, resulting in antibacterial and antifungal properties [1–6]. Earwax usually migrates out of the ear canal via natural jaw actions, such as chewing and talking, but in some cases, it can accumulate excessively, leading to occlusion within the external ear canal. This causes various unwanted symptoms, the most common being hearing loss, with other self-reported symptoms such as tinnitus, a blocked ear, changes in the quality of one’s own voice, pain or discomfort, coughing, irritation, infections and even dizziness [1, 3, 5, 7–16]. Munro et al. [16] reported that the most common symptom of earwax was hearing loss (86.4% of patients), which affects daily living, including listening to TV and environmental awareness. Occluding earwax can also have a negative effect on a person’s quality of life by causing social withdrawal, reduced function at work, mild paranoia and disorientation [1, 11, 14].

The prevalence of problematic earwax is reported to be approximately 2.3 million people per year, or 5% of the general adult population, which is as high as 65% of the elderly population [1, 11]. Occluding wax will only clear spontaneously in 5% of cases, which explains why earwax removal, which is the most common ENT procedure undertaken in primary care, is performed on 4 million ears per year in the UK with the goal of resolving or reducing these unwanted symptoms [1, 12, 17].

#### Rationale

Despite earwax removal being a relatively common procedure, there is limited high-quality evidence on the most effective removal method. Many studies recommend the use of a wax softener prior to mechanical removal [18]. Wax softeners have been used for many years in all healthcare settings for the management of wax as a pretreatment prior to mechanical removal [2, 18–20]. There are various pretreatment wax softeners available, and studies have shown no difference in the use of olive oil, water or any other wax softener [1, 13, 19–21]. Using a wax softener alone may lead to complete clearance of the wax in approximately 20–22% of cases, but most often, it needs to be combined with mechanical removal [13, 19, 22, 23].

The NHS Audiology Primary Care service in North Wales offers a wax removal service located at GP practices. This is a needs-based pathway in which patients reporting symptoms consistent with excessive earwax can self-refer, or patients can be referred by professionals who confirm the presence of earwax that restricts a full view of the tympanic membrane for earwax removal. All patients are advised to use olive oil drops or spray as a pretreatment wax softener followed by attempted earwax removal via microsuction by audiology practitioners as a first-line treatment. The service evaluation data revealed that one in four patients required a return visit for a second attempt at wax removal, with the first attempt being unsuccessful due to incomplete removal of wax, as reported by the audiology practitioner. Anecdotal evidence from our practitioners suggests that administering olive oil as a spray may lead to an improved success of wax removal compared with the traditional method of administering drops. This may be due to more effective coverage and contact with the wax, which is in agreement with Oron et al. [24], who reported that spray may penetrate deeper into the ear canal during application.

This large-scale cluster randomised control trial aims to compare the administration methods of olive oil drops and olive oil spray as a pretreatment wax softener prior to wax removal by microsuction. No previous study has investigated whether the application of wax softeners via different methods affects the outcome of wax removal. The trial has full support from a public and patient involvement (PPI) group who have been consulted throughout the trial design and who will also be involved in the analysis and dissemination of results. PPI individuals reported this research to be valuable and important, especially within the theme and focus on preventative care and care in the community.

### Objectives {7}

Primary objective: To compare the success of wax removal following the use of olive oil pretreatment with wax softener intervention (spray) and comparator (drops) for 7 days prior to microsuction. The success of wax removal is determined by complete clearance of wax not requiring a second visit to the clinic, as reported by the audiology practitioner upon otoscopic examination following microsuction.

Secondary objectives:

The secondary objective outcomes will be compared between the olive oil drops and olive oil spray groups.

- To explore improvements in self-reported symptoms following wax removal, a self-reported improvement scale is used.
- To investigate the postmicrosuction visualisation ratings (a measure of the amount of residual wax).
- The occurrence of adverse events associated with microsuction are monitored according to Clegg et al. [25].

### Trial design {8}

**Fig. 1:**
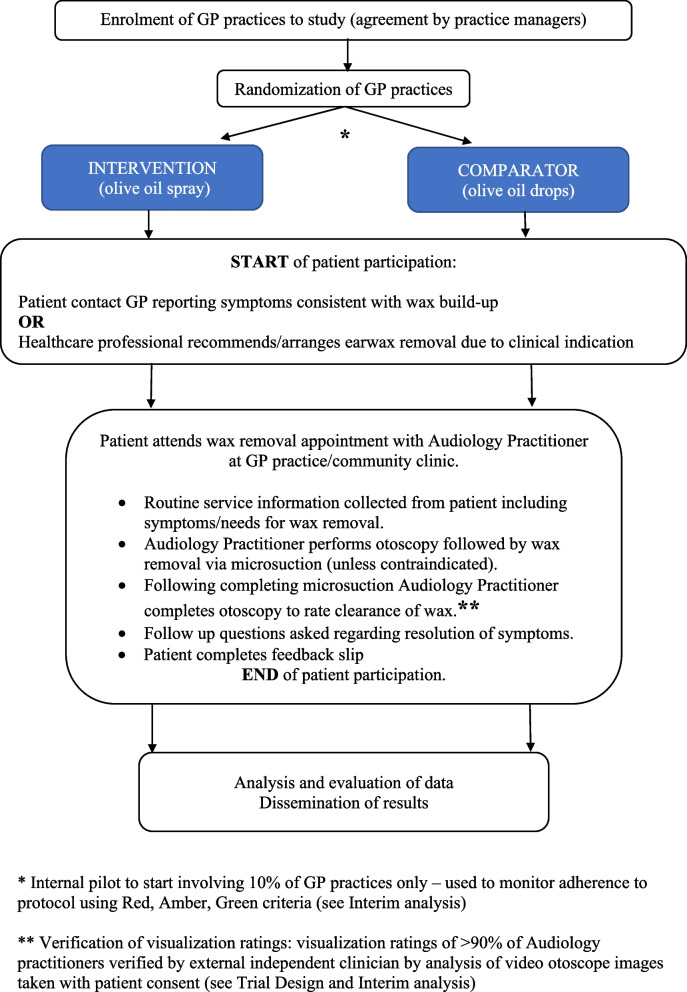
RCT flowchart

This RCT is a parallel, two-arm, superiority cluster randomisation trial design to compare the effectiveness of olive oil drops and olive oil spray when used as a pretreatment wax softener prior to wax removal by microsuction. A cluster randomised design was chosen to ease the organisation of the trial, as it takes place within a current clinical service, and individual randomisation would add to the workload and make the trial difficult to undertake. Twenty-six GP practices will be included and randomised via a one-to-one allocation ratio to either the intervention arm (olive oil spray) or the comparator arm (olive oil drops) prior to the start of the study. The trial is designed to have a pragmatic approach, with the data analysed via an intention-to-treat analysis. Figure 1 presents the trial flow chart. Minimal changes are introduced to the service as part of this trial but include:

- Verification of one of the secondary outcomes, which involves reporting the amount of residual wax following microsuction by the audiology practitioner. The amount of residual wax following microsuction is recorded by the audiology practitioner via a visual verification rating (0–3); see Table 1. A video otoscopy image will be taken with patient consent and sent to an independent professional, who will rate the amount of residual wax using the verification ratings. The ratings of the audiology practitioner and independent professional will be compared to verify the reliability of this outcome.
- Patients will be given a feedback slip at the end of the appointment with the audiology practitioner and asked to select which pretreatment wax softener they used. This information will be used to check adherence to the protocol when analysing the outcomes given that the pretreatment wax softener is purchased and self-administered by the patient.

**Table 1:** Descriptors for visualisation ratings used postmicrosuction by audiology practitioners

Visualisation rating	Explanation
0	No or very minimal earwax, complete visualisation of tympanic membrane
1	Minor amount of earwax with majority of tympanic membrane visible
2	Moderate amount of earwax with only part of the tympanic membrane visible
3	Fully occluding wax, no visualisation of tympanic membrane

## Methods: participants, interventions and outcomes

### Study setting {9}

The study will run within 26 NHS-funded GP practices that deliver the NHS Audiology Primary Care wax removal service across North Wales. The earwax removal service is delivered by audiology practitioners. Patients with symptoms, or a clinical need, can self-refer, be referred by their audiology practitioner, GP or other healthcare professional working in the practice. The research will not change service provision or routine clinical care for research participants or other patients seen within the service during the research timeline.

### Eligibility criteria {10}

All adults over 18 years of age with the presence of earwax in one or both ears are eligible for the study.

Inclusion criteria:

- Adults over 18 years of age
- Provide informed consent for microsuction
- Presence of wax given rise to symptoms or need that warrants removal

The exclusion criteria include:

- Under 18 years of age
- Active ear infection
- Active dermatitis
- Perforated or nonintact tympanic membrane
- Foreign bodies present in the ear canal
- Any contraindication for microsuction or use of wax softeners – previous ear surgery including mastoid obliteration; grommets in situ

### Who will provide informed consent? {26a}

Consent to participate in this trial will be presumed, as the study participants in both arms are NHS patients accessing standard NHS care, and this approach has been approved by ethics. The only difference between the two arms is that patients will be advised to use either pretreatment olive oil drops or spray rather than having a free choice of using either solution.

Written consent will be obtained from a selection of patients as part of the verification of visualisation ratings for outcome verification (see Fig. 1). Five patients seen by 90% of the audiology practitioners will be asked to provide consent for a video otoscopy image of their external ear canal. The CI will obtain informed consent and take video otoscope images. This image will be shared with an independent professional for outcome verification purposes. A copy of these signed and dated forms will be kept in the patient’s audiology records, and another file with trial documentation will be included in the research file. These will not be shared with anyone outside the research team.

### Additional consent provisions for the collection and use of participant data and biological specimens {26b}

Not applicable – There are no additional consent provisions for sharing data with others in this trial.

## Interventions

### Explanation for the choice of comparators {6b}

The comparator in this trial is olive oil drops, which are traditionally the most commonly used pretreatment wax softener within our wax removal service. The olive oil will be self-administered at home by the patient or a significant other at a recommended dose of 3 drops per ear once a day for 7 days prior to the wax removal appointment. Following feedback from the PPI individuals, advice was given by a Trial Pharmacist, who confirmed that olive oil sold for wax removal purposes in pharmacies has been through medical-grade testing rather than ordinary kitchen olive oil, so this is recommended for use as part of this trial.

### Intervention description {11a}

The intervention in this trial is olive oil spray. Patients will be advised to use the allocated olive oil solution when booking their wax removal appointment at the GP practice. The olive oil will be self-administered at home by the patient, or a significant other, at a recommended dose of 3 sprays per ear once a day for 7 days prior to the wax removal appointment. Notably, olive oil spray has a greater cost than does the comparator. This was discussed with the PPI individuals, who deemed the price difference acceptable and unlikely to influence many participants.

### Criteria for discontinuing or modifying allocated interventions {11b}

All patients will be encouraged to follow the pretreatment advice as part of this trial. The option to discontinue or modify the pretreatment advice will be at the discretion of the healthcare practitioner should this be clinically indicated.

### Strategies to improve adherence to interventions {11c}

Patient information leaflets are available at participating GP practices that specifically instruct patients to administer pretreatment olive oil as per the allocation of the GP practice. The patient information leaflet highlights that the research is being carried out to investigate any differences between these two administration methods, hence the importance of adhering to the intervention.

Staff reminder slips will be issued to GP practices to be placed in reception and administration offices. Additionally, staff members will be reminded regularly of the importance of encouraging patients to use the allocated solution both by the research team and the Audiology Primary Care team.

Following PPI feedback regarding the occasional lack of stock of olive oil spray in community pharmacies, local community pharmacy networks have been informed of the research to encourage local pharmacies to keep stock of both drops and sprays for patients to purchase locally.

### Relevant concomitant care permitted or prohibited during the trial {11d}

Patients may be offered concomitant care for hearing or balance assessment alongside this trial if needed.

Patients will be given verbal pre-appointment instructions when booking their wax removal appointment to use the olive oil drops or spray solution. They will be advised to use three drops or sprays of the olive oil, once a day for seven days prior to their wax removal appointment. Written information will be available for those that arrange their wax removal appointment whilst in the GP practice. The patient information leaflet specifies to use olive oil drops or spray only (depending on cluster allocation) and not any other available wax softener. These instructions will be given verbally to those patients who arrange their wax removal appointment by phone.

### Provisions for posttrial care {30}

Not applicable to this trial, as patients will only remain in the trial for the duration of one clinical visit, and there are no other deviations from normal clinical care.

### Outcomes {12}

#### Primary outcome

The primary outcome is a comparison of the success of wax removal across the two groups. Success of wax removal is measured by the Audiology Practitioner, who inspects the ear and calculates the proportion of patients where wax removal is successful (0 or 1 as defined in Table 1) at the first attempt and who do not need a repeat visit (2 or 3 as defined in Table 1).

#### Secondary outcomes

The secondary outcomes include the following:

- Improvements in self-reported presenting symptoms (reported by patients during clinic visits prior to microsuction) measured via a 5-point improvement rating scale (much better, slightly better, about the same, slightly worse, and much worse) following wax removal in the two groups.
- Differences in the amount of residual wax following microsuction are measured by a postmicrosuction visualisation rating reported by the audiology practitioner through visual inspection of the ear. See Table 1.
- Adverse events. All adverse events that occur in the clinic during or immediately after microsuction and any reported by patients up to one week after microsuction, will be recorded as per normal clinical practice.

A further exploratory analysis will be completed on the following:

- The effect of age on the success of wax removal.
- Success of wax removal in patients who do not use any pretreatment.

### Participant timeline {13}

Patients are involved in the study for wax removal. The trial requires one clinic visit for every patient. Patients will be advised to use the allocated intervention for seven days prior to their appointment and then will visit the clinic for the wax removal appointment. This is shown in the trial flow chart (see Fig. 1).

### Sample size {14}

A total sample size of 1,742 participants across 26 clusters/GP practices (67 participants in each practice) was calculated as sufficient to detect a minimal clinically important difference of 9%, with an 80% power level and 5% significance. The sample size for this trial was determined via ‘The Shiny CRT Calculator’ (accessible here: https://clusterrcts.shinyapps.io/rshinyapp/lculator: Power and Sample size for Cluster Randomised Trials (shinyapps.io)). The following parameters were used (trial design = parallel; correlation structure = exchangeable; differential clustering = false; allowance for varying cluster sizes = yes; coefficient of variation = 0.527; number of clusters per arm = 13; intracluster correlation = 0.02; ICC lower extreme = 0.01; ICC upper extreme = 0.05; outcome type = binary; proportion under control = 0.74; proportion under investigation = 0.83).

The above parameters were determined via discussion with statisticians and by professional consensus. A consensus was reached among 13 professionals regarding the minimal clinically important difference in the use of a specified pretreatment wax softener. The majority of the responses from the 13 professionals were between 6 and 10%, so for a conservative approach, we decided to use 9%, which was also the average.

The NHS Audiology wax removal service in North Wales is still in the process of roll out, and the actual number of GP practices participating in the study may be greater than 26. If this is the case, the sample size calculation will be reviewed.

### Recruitment {15}

The participants will be individual NHS patients who access the earwax removal service. They will be identified by the GP surgery administration teams or healthcare staff as per usual practice (patient contacts or is advised by healthcare professionals to request an appointment for wax removal or reporting symptoms/needs that are thought/confirmed to be because of wax build-up). The normal procedure for booking a wax removal appointment will be followed, and staff at the GP practice will be fully informed of the research. When booking the appointment, patients will be informed of the pretreatment they should use for seven days prior to their wax removal appointment by the staff member booking their appointment. Patients can book their appointment face-to-face whilst visiting the surgery or by telephone. Patients will be offered written information about using the pretreatment and will be directed to a video to watch if needed. Patients will be advised to purchase their olive oil drops or olive oil spray from a pharmacy as usual clinical care.

## Assignment of interventions: allocation

### Sequence generation {16a}

Randomisation will take place at a cluster level (GP practice) with a one-to-one allocation ratio to the two groups of olive oil drops or olive oil spray via stratified block randomisation. Stratifying was performed on the basis of site size (small, large) with block sizes of 2 and 4. A total of 26 clusters will be randomised. Randomisation is performed via the randomisation software Sealed Envelope.

### Concealment mechanism {16b}

The CI will implement the allocation sequence and inform each GP practice of their allocation via email and personal visits. All precautions will be taken to conceal the randomisation allocation from the audiology practitioners delivering the earwax removal service, but patients may accidentally disclose this, or practitioners may be able to identify the pretreatment product used on the basis of otoscopy findings. Throughout the research, each GP practice is identified and allocated by a unique numerical code only known by the CI.

### Implementation {16c}

The CI will generate the allocation sequence, enrol GP practices and assign the interventions to the clusters (GP practices). As a pragmatic trial, the research is happening within normal clinical practice; therefore, patients are not officially enrolled in the study, but rather, the GP practice staff (receptionist, healthcare staff) will inform the patient of the research when booking in for the wax removal appointment. The GP practice staff will be responsible for informing the patients of the allocated intervention (olive oil drops or spray).

## Assignment of interventions: blinding

### Who will be blinded {17a}

Care providers (audiology practitioners) and the outcome verification professional will be blinded to the assignment of interventions, but we cannot accommodate patients accidentally disclosing this information during their appointment. The majority of audiology practitioners see patients from various GP surgeries at one or more locations, meaning that they are likely to see patients who have been allocated to both the comparator and the intervention during a single clinical session. Only the CI will be aware of the complete assignment of interventions, and the primary care audiology clinical leaders and GP staff members at each individual practice will be aware of their own assigned intervention. It is not possible to blind the trial participants to the assigned intervention.

### Procedure for unblinding if needed {17b}

There is no requirement for a procedure for unblinding during this trial, as there is no risk associated with the blinding, as both interventions are the same solution only delivered in a different manner.

## Data collection and management

### Plans for assessment and collection of outcomes {18a}

The trial will use routine clinical data collected from patients accessing the audiology wax removal service. No additional data are collected as part of this research, which is not routinely collected for service evaluation. No patient identifiable data are collected, and all data are recorded at the microsuction appointment visit. The premicrosuction data recorded included date, location, age, referral source and self-reported presenting symptoms. The postmicrosuction data recorded included the amount of residual wax in each ear (visualisation rating) and the degree of improvement in self-reported presenting symptoms using a 5-point scale (much better, somewhat better, no change, somewhat worse, much worse). All the data are recorded during a single clinic visit.

All the data collected will be recorded on a password-protected Excel spreadsheet currently used by audiology practitioners for evaluation purposes. The spreadsheet is kept online on the Audiology SharePoint intranet site, meaning that it is accessible to all audiology practitioners working at various NHS locations.

Only patients with a complete outcome dataset were included in the final analysis.

### Plans to promote participant retention and complete follow-up {18b}

Information about the research trial is included on the NHS audiology service’s external website and as posters in the participating GP practices to raise awareness.

If patients report that they have not complied with advice and have not used a pretreatment wax softener, the audiology practitioner will continue to attempt to remove any wax via microsuction via routine service delivery. The data for these patients will be analysed separately from those for the other patients in an exploratory analysis.

### Data management {19}

All research data will be recorded and held on a password-protected Excel spreadsheet on the internal NHS Audiology services’ online SharePoint site, as is the current practice for service evaluation. The data will be accessible by all audiology practitioners working at the wax removal service, their service leads and the CI of the trial. There will be a focus on ensuring that the data are accurate, eligible, complete, consistent and available when needed, hence the reason for it being kept in an easily accessible location. The CI will extract the required data from the source file for analysis purposes. The data will not be shared with anyone outside the research team at any time.

A numerical identification code will be allocated to the selection of patients who consent to a video otoscopy image of their ear following microsuction. This code will be added to their corresponding data input on the main spreadsheet by the CI and on their photographic consent form. The numerical code and corresponding consent form with the patient’s name will only be available and known by the CI.

Patients will have the opportunity to share their email address to receive a summary of the trial outcomes. Their email address will not be able to be linked back to their research data entry and will be shared only with the CI. The email address will be recorded on individual GP practice patient slips alongside a GP clinic code related to the research allocation. The GP clinic codes and allocated treatment details will be stored electronically on the internal NHS Audiology online Sharepoint site in a password-protected Excel spreadsheet accessible only by the CI. This will be deleted at the end of data collection.

Both the written video otoscopy consent forms and the GP practice patient slips with completed email addresses will be stored in the research file within a locked cabinet that will be accessible only by the CI.

In compliance with good clinical practice (GCP), all documentation relevant to the trial will be retained for a period of 5 years following the end of the RCT. Further information on data management can be found within the protocol.

### Confidentiality {27}

No patient identifiable information is collected as part of this research study apart from the individuals who are selected and consent to the video otoscopy verification exercise and those who volunteer to share their email address at the end of the clinic visit to be informed of the research outcome. Both the photography consent forms and completed GP practice patient slips with email addresses will be stored in the research file in a locked cabinet at the main NHS hospital site, accessible only by the CI. Any link between codes and identifiers on consent forms will be deleted at the end of data collection.

### Plans for collection, laboratory evaluation and storage of biological specimens for genetic or molecular analysis in this trial/future use {33}

Not applicable – no biological samples are collected or evaluated in this trial.

#### Patient and public involvement

A group of five patient and public volunteers was consulted during the protocol design stage. The CI conducted two focus groups that discussed the acceptability of the research, the methodology used and the data collected. All volunteers agreed the research was worthwhile especially as the outcome may benefit patient care by potentially reducing repeated clinic visits for wax removal. They reported the methodology was acceptable and specifically supported the concept of not changing clinical practice for research purposes. Their feedback also influenced the content of the patient information material used in the study. The volunteers suggested, and we agreed to, include information on: (i) the specific type of olive oil that should be used (e.g. olive oil sold in pharmacies as opposed to ordinary ‘kitchen’ olive oil) and (ii) the number of applications of the olive oil per day. Their feedback also led to the inclusion in the protocol of a list of factors that were assumed to be random across the two groups – ethnicity, olive oil administration differences (e.g. self or assisted administration), duration of wax problems, and prior experience of wax softener use. We agreed to provide the volunteers with a summary of findings on completion of the study.

## Statistical methods

### Statistical methods for primary and secondary outcomes {20a}

Analysis and reporting of the trial will follow the Consolidated Standards of Reporting Trials (CONSORT) guidelines for cluster trials [26].

For the primary outcome analysis, a mixed effects logistic regression model will be fitted to the data with a random intercept for cluster and a fixed effect for wax softener delivery type (olive oil drops or olive oil spray) and strata (small or large GP practice). A correction factor is applied to reduce the risk of inflating the type I error rate due to the small number of clusters (< 40).

For the secondary outcomes investigating visualisation ratings and improvement in self-reported symptoms, we will use a linear mixed effects ordinal regression model with a random intercept for both clusters and patients and a fixed effect for wax softener delivery type (olive oil drops or olive oil spray) and strata (small or large GP practice). To investigate whether the reported improvement in self-reported symptoms/needs of patients who used olive oil drops significantly differed from that of patients who used olive oil spray, the unit of analysis is patient. To analyse whether there was a difference in the amount of residual wax measured by visualisation ratings between the olive oil drops and spray groups, the unit of analysis is the ear. The number of adverse events reported in the two groups will be analysed descriptively because of the likely low number of adverse events. Further details of the statistical analysis plan can be found within the protocol.

### Interim analyses {21b}

The trial includes an internal pilot that involves conducting the study as planned within a sample (10%) of the GP practices. Data will be collected as per the full study, and the Red, Amber, and Green criteria will be used. If there is less than 50% compliance (red) with the protocol, data collection will stop, and urgent training visits will be arranged to ensure that patients receive appropriate advice from healthcare professionals on the use of the allocated intervention. When compliance is between 50 and 70% (amber), data collection may be paused temporarily, and GP practices may be in contact with a research reminder. If compliance is 80% (green), data collection will continue.

A planned interim analysis involves verifying the visualisation ratings following microsuction. The audiology practitioner uses a numerical visualisation rating to categorise how much wax remains in the ear following a microsuction attempt. To verify these ratings, an anonymised photograph of the external ear canal and ear drum will be taken via a video otoscope with individual patient consent. The image is saved via a numerical code that is related to the GP practice and the age of the patient. The image is then shared with an independent experienced professional who views the image and records an independent visualisation rating. This rating will be compared with the audiology practitioner’s rating, and a Red, Amber, Green strategy (as above) will be used to validate the agreement in ratings.

There are no other formal stopping rules for the trial outside of the initial internal pilot (as described above).

### Methods for additional analyses (e.g., subgroup analyses) {20b}

Exploratory analysis of the effect of age will be performed by rerunning the primary and secondary analyses with an additional covariate of the fixed effect of age in the multilevel model.

Additionally, we expect that some patients will not adhere to the protocol and will not use any pretreatment wax softeners before attending their wax removal appointment. We will repeat the primary and secondary analyses and add a covariate fixed effect of no pretreatment used in the multilevel model. Further details can be found in the protocol.

### Methods in analysis to handle protocol nonadherence and any statistical methods to handle missing data {20c}

A sensitivity analysis will be run by removing the patients who did not use pretreatment and rerunning the analyses of the primary and secondary outcomes. As all the data are nonidentifiable, there will be no way of identifying individual patients who have not adhered to the protocol. Only patients with a full dataset were included in the analysis.

### Plans to give access to the full protocol, participant-level data and statistical code {31c}

Anonymized data can be made available from the authors following reasonable request upon completion of the trial.

## Oversight and monitoring

### Composition of the coordinating centre and trial steering committee {5d}

The day-to-day running of the trial is coordinated and monitored by the CI with support from the protocol contributors and the three BCUHB Primary Care Audiology Clinical Leads who lead the wax removal service. The CI will have oversight and ongoing monitoring during data collection. The CI and two protocol contributors make up the Trial Management Group who meet monthly to discuss and monitor overall trial progress. Trial progress is then reported to the Sponsor representative by the CI. There is no formal Trial Steering Committee associated with this trial.

### Composition of the data monitoring committee, its role and reporting structure {21a}

A Data Monitoring Committee was not deemed necessary for this trial due to it being a low-risk study. The trial is run within an existing clinical service for which the CI will monitor on an ongoing basis.

### Adverse event reporting and harms {22}

Adverse events are not anticipated during this trial, as the two interventions are routinely used. Unexpected severe adverse events (SAEs) associated with the standard care intervention (ear wax removal) will be reported to the sponsor as per our Standard Operating Procedure for Safety Reporting and further assessed by the CI in addition to following standard clinical care procedures. Non-severe adverse events will be recorded, assessed, reported and managed as per normal service delivery and departmental policy and as stated in the research protocol.

### Frequency and plans for auditing trial conduct {23}

The trial conduct will be monitored by a sponsor-appointed monitor (BCUHB Research & Development Deputy Manager) throughout the trial life cycle according to the sponsor risk assessment and a trial-specific monitoring plan per standard operating procedures. The Trial Management Group will meet monthly to review trial progress and conduct. The study was deemed low risk meaning a Data Monitoring Committee was not deemed necessary; however, it may be included in routine audits conducted by the sponsor according to the sponsor audit schedule.

### Plans for communicating important protocol amendments to relevant parties (e.g., trial participants, ethical committees) {25}

Protocol amendments will require local approval by the sponsor, ethics committee and healthcare research workers (HCRWs) before implementation. Once approved the amended protocol will be added to the Investigator Site File and will then be shared with all participating GP practices, the audiology team delivering the wax removal service, investigators, RECs, trial registries, journals and research sponsors. The protocol on the clinical trial registry will also be updated. Any deviations in protocol during the study will be fully documented and reported using a breach report form.

### Dissemination plans {31a}

Trial patients have the opportunity to note their email address if they would like to hear about the trial outcomes. At the end of the trial, a summary of the research and the outcome will be shared with all patients who opt to share their email addresses.

The research and outcomes will be published nationally in a peer-reviewed journal and at national/international conferences. The findings will be shared within the field of audiology and further within general healthcare networks. We are investigating ways to minimise barriers to the implementation of research findings by involving healthcare practitioners who will be providing this advice to patients in this trial (GP practice staff). Should the research show that either the intervention or comparator is more effective as a pretreatment wax softener, this information will be used to change recommendations to patients at all Wales service levels following approval from the Wales Audiology Heads of Service Group. The information will also be shared with the British Society of Audiology as new evidence for consideration to inform an update to the Aural Care (Ear Wax Removal) Practice Guidance and shared with NICE to supplement future recommendations for wax management.

## Discussion

This research aims to answer an important question, as deemed by PPI individuals, that has the potential to impact national guidance on wax removal procedures. This pragmatic RCT runs within a current clinical service and has been designed to reflect real-life clinical practice and minimise disruption to clinical services. Audiology practitioners at any level do not currently have prescribing rights; thus, this trial is restricted by an inability to supply the olive oil solution to patients, meaning that we have adhered to the current clinical procedure in that patients source the olive oil solution themselves. This does carry a potential risk of patients not adhering to the protocol owing to product availability, issues sourcing the solution locally or failure to follow clinical recommendations. Following PPI feedback, we have attempted to minimise this by contacting community pharmacy teams across North Wales, informing them of the research and advising them to stock both olive oil drops and olive oil spray in their pharmacies for the benefit of this research and by using patient information materials and posters that highlight the importance of this research.

The data will be analysed via an intention-to-treat analysis. This may mean that any difference between the two groups will be minimised, indicating that the pragmatic approach taken with the aim of better reflecting a real-life clinical situation may be seen as a strength of the trial. An alternative approach would need to be highly regulated, and any findings may not necessarily be effective in real life.

Data collection is to be completed by audiology practitioners, and no additional data are being collected compared with those already being collected for data evaluation purposes. We hope this will help ensure that there are no missing data entries. All audiology practitioners involved in the service will be aware of the RCT, and its objectives and clinical leaders of the service will be involved and approved for the study design.

The findings of this research will only be applicable to the use of pretreatment with the wax softener olive oil followed by the mechanical removal method of microsuction. Further research will be needed to determine whether the same findings apply to other pretreatment wax softeners or mechanical removal methods, such as irrigation.

The research is run within 26 sites across North Wales. While the CI will maintain close contact with all the sites during the research, the risk remains that primary care staff booking the wax removal appointment and advising patients on the use of pretreatment wax softeners may not give the correct advice or may not emphasize the importance of adhering to recommended guidance. To help support this, each cluster site has been issued staff reminder slips that are displayed behind reception and in staff areas as reminders of the advice that should be given to patients.

PPI representatives were included in the development of the study design through focus groups and email correspondence. Their input shaped the design of the protocol and methodology by validating the design and informing protocol details such as the difference between olive oil sold in pharmacies for wax removal purposes and ordinary kitchen olive oil and acceptance of price differences to patients between the investigator and comparator product. All PPI individuals approved the trial as important research and supported the ethical approval process. The PPI individuals will be kept informed of the progress of the trial as the research progresses.

## Trial status

Protocol version 2.3.

26th August 2025.

Recruitment for the study began on 1 st January 2025, and we approximate the end of data collection on 31st December 2025.

## Supplementary Information

Supplementary Material 1.

## Data Availability

Anonymized data following the end of the research study will be authorized by the sponsor. The research team will be responsible for archiving all video otoscopy consent forms and GP practice patient slips that contain participant email addresses and clinical data records. These records and anonymized data will be retained for a minimum of 5 years after completion of the trial.

## Abbreviations

AE: Adverse event
BCUHB: Betsi Cadwaladr University Health Board
CI: Chief investigator
ENT: Ear, nose and throat
GCP: Good clinical practice
GP: General practice
HCRW: Health Care Research Wales
NHS: National Health Service
PPI: Patient and public involvement
REC: Research Ethics Committee
R&D: Research and development
SAE: Serious adverse event
