# Supplementary material for: A comparison of the clinical effectiveness of pretreatment olive oil administered as drops versus spray prior to earwax removal by microsuction in adults: a protocol for a cluster randomised control trial
Source: Trials. 2025 Dec 18;27:79. doi: 10.1186/s13063-025-09378-5 (PMC12831375; doi:10.1186/s13063-025-09378-5)
Supplement: Supplementary file 1 — Supplementary Material 1. [file 13063_2025_9378_MOESM1_ESM.docx]

SPIRIT figure - A comparison of the clinical effectiveness of pretreatment olive oil administered as drops versus spray prior to earwax removal by microsuction in adults: a protocol for a cluster randomised control trial.

|  | STUDY PERIOD | | | | | | | | | |  |  |  |  |  |  |
| --- | --- | --- | --- | --- | --- | --- | --- | --- | --- | --- | --- | --- | --- | --- | --- | --- |
|  | Enrolment | Allocation | Data collection period | | | | | | | | | | | | | End of study |
| TIMEPOINT | Nov 24 | Dec 24 | Jan 25 | Feb 25 | Mar 25 | Apr 25 | May 25 | Jun 25 | Jul 25 | Aug 25 | Sep  25 | Aug  25 | Oct  25 | Nov  25 | Dec  25 | Jan  26 |
| Enrolment of GP practices (clusters) | X |  |  |  |  |  |  |  |  |  |  |  |  |  |  |  |
| Allocation/  Randomisation |  | X |  |  |  |  |  |  |  |  |  |  |  |  |  |  |
| Recruitment of patients (self-administration of intervention and comparator) |  |  |  |  |  |  |  |  |  |  |  |  |  |  |  |  |
| Data analysis |  |  |  |  |  |  |  |  |  |  |  |  |  |  |  | X |
